# Human Papilloma Virus Increases ΔNp63α Expression in Head and Neck Squamous Cell Carcinoma

**DOI:** 10.3389/fcimb.2020.00143

**Published:** 2020-04-08

**Authors:** Simona Citro, Alice Bellini, Alessandro Medda, Maria Elisa Sabatini, Marta Tagliabue, Francesco Chu, Susanna Chiocca

**Affiliations:** ^1^Department of Experimental Oncology, IEO, European Institute of Oncology IRCCS, Milan, Italy; ^2^Division of Otolaryngology Head & Neck Surgery, IEO, European Institute of Oncology IRCCS, Milan, Italy

**Keywords:** head and neck cancer, head and neck carcinoma, HPV, HPV16, p63

## Abstract

P63, and in particular the most expressed ΔNp63α isoform, seems to have a critical role in the outcome of head and neck cancer. Many studies have been conducted to assess the possible use of p63 as a prognostic marker in squamous cell carcinoma cancers, but the results are still not well-defined. Moreover, a clear relationship between the expression of ΔNp63α and the presence of high-risk HPV E6 and E7 oncoproteins has been delineated. Here we describe how ΔNp63α is mostly expressed in HPV-positive compared to HPV-negative head and neck cancer cell lines, with a very good correlation between ΔNp63α mRNA and protein levels.

## Introduction

The p53 family of transcription factors, including p53 (TP53), p63 (TP63), and p73 (TP73), are key players in tumor development and formation (Vousden and Prives, 2009). While the p53 gene is mutated or lost in the majority of human cancers, neither p63 nor p73 show frequent somatic mutations in neoplasia (Deyoung and Ellisen, 2007). All three p53 family members encode proteins with strong homologies to p53, not so much in terms of primary sequence, but in overall domain structure and conformation, with high homology in the DNA-binding domains. p63 and p73 are expressed from two distinct promoters, which produce two isoforms either containing or lacking the N-terminal transactivation domain (TAp63/p73 and DNp63/p73, respectively) (Deyoung and Ellisen, 2007). Alternative splicing of the 3′ end of the TA and ΔNp63 mRNAs produces the α, β, and γ isoforms. ΔNp63α is the most abundant isoform detected in the basal layer of mucosa, skin, and other epithelial tissues and it is overexpressed in up to 80% of primary Head and Neck Cancers (HNC).

During development, ΔNp63α expression is restricted to epithelial stem cells and the undifferentiated basal layer of stratified epithelia and is critical for the development and maintenance of stratified epithelial tissues (Oh et al., 2011). Nevertheless, ΔNp63α functions as a potent oncogene in squamous cell carcinomas (SCCs) of diverse origins (Rocco et al., 2006; Yang et al., 2011). In particular, we have recently shown how ΔNp63α is critical for cellular proliferation and migration in HNC cell lines (Citro et al., 2019).

HNC affect ~600,000 patients per year worldwide and is the sixth leading cancer by incidence. Smoking is mainly implicated in the rise of HNC in developing countries, while the human papillomavirus (HPV) is an established important risk factor in the increase of oropharyngeal cancers in developed countries (Ang et al., 2010; Sabatini and Chiocca, 2019; Sandulache et al., 2019).

Although HPV-positive and HPV-negative HNC are characterized by etiological, biological and clinical heterogeneity, the same therapeutic protocol, comprising surgery, radiation and chemotherapy, is used. In this study we characterized a different pattern of expression of ΔNp63α in HNC cell lines, comparing HPV-positive and HPV-negative HNC cell lines. We clearly show an increase of both ΔNp63α mRNA and protein levels in HPV-positive compared to HPV-negative cell lines, which is dependent on the presence of the HPV E6 and E7 oncoproteins.

## Methods

### Cell Culture

HNC cell lines were acquired from different sources (Brenner et al., 2010) and were already accurately described and cited (Steenbergen et al., 1995; Ballo et al., 1999; Ragin et al., 2004; White et al., 2007; Brenner et al., 2010; Tang et al., 2012) in our previous study (Citro et al., 2019). Every 6 months all cell lines were authenticated by short tandem repeat profiling and tested for mycoplasma contamination. Skin biopsies were collected via standardized operative procedures approved by European Institute of Oncology Ethical Board. Informed consent was obtained from all patients (donors). Adult human epidermal keratinocytes (HKs) were prepared and maintained as previously described (Pozzebon et al., 2013; Mattoscio et al., 2017). Briefly, skin biopsies from donors were digested with Dispase (10 U/mL; Gibco) for 4 h at 37°C to remove the epidermis, followed by a trypsinization step (Trypsin 500 mg/L) for 30 min at 37°C to obtain isolated cells. Primary cultures of the isolated cells were then maintained in Keratinocyte Serum-Free Medium (KSFM; Gibco) containing bovine pituitary extract (BPE, 30 μg/mL; Gibco) and epidermal growth factor (EGF, 0.2 ng/mL; Gibco). Cells from passages 2–5 were used for the experiments. All cells were cultured at 37°C in a 5% CO_2_ buffered incubator.

### Transduction, Transfection, and Plasmids

pLXSN HPV16E6/E7 and empty vector were previously described (Mattoscio et al., 2017). For retroviral transduction, plasmids were transfected into Phoenix Ampho cells by calcium-phosphate method. The following day, HKs were transduced with retroviral supernatants for 6 h at 37°C for 2 days and selected with G-418 Sulfate (Gibco) for 1 week and finally collected for RNA extraction or WB (Mattoscio et al., 2017). HPV-positive HNC cell lines were transfected with Lipofectamine 2000 (Thermo Fisher Scientific) with siLuciferase or E6/E7 siRNAs (Mattoscio et al., 2017) following manufacturers' instruction. 72 h after transfection, cells were collected for WB analysis.

### Cell Lysis and Western Blot

Cells were lysed in E1A buffer (50 mM HEPES pH7, 250 mM NaCl, 0.1% NP-40, proteases inhibitors, 0.5 mM NEM, 0.5 mM NaF, and 2 mM Na_3_VO_4_). After lysis, an equal amount of protein for each sample was resuspended in denaturing sample loading buffer, separated on SDS-PAGE gel and immunoblotted with the indicated antibodies. The following antibodies were used: p63 (mouse, Abcam), HPV16 E7 and p53 (mouse, Santa Cruz), and Vinculin (mouse, Sigma Aldrich) as loading control. Membranes were then incubated with the appropriate horseradish peroxidase (HRP) secondary antibodies and the signal was acquired with Chemidoc (Bio-Rad).

### RT-qPCR

RNA was extracted from cells with the Quick-RNA MiniPrep kit (ZYMO RESEARCH). cDNA was generated by reverse transcription-PCR with Reverse Transcriptase (Promega). Relative levels of specific mRNAs were determined with the Fast SYBR Green detection chemistry system (Applied Biosystem). All PCR reactions were performed with a 7500 Fast Real-Time PCR system (Applied Biosystem). Ribosomal Phosphoprotein (RpPO) was used as a house-keeper gene for normalization. Densitometric analysis of the intensity of the protein bands were preformed using ImageJ software (Rasband W.S., ImageJ, U. S. National Institutes of Health), and standardized to the housekeeper levels.

### Statistical Analysis

Statistical differences were evaluated using unpaired *t*-test (Graphpad Prism version 6 software).

## Results

### HPV-Positive HNC Cell Lines Expressed Higher Protein Level of ΔNp63α Compared to HPV-Negative Cell Lines

**Figure 1:**
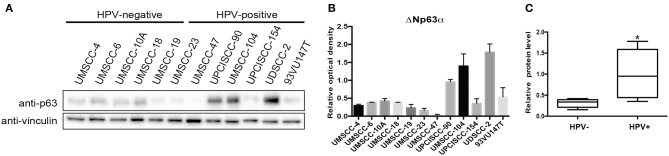
ΔNp63α protein levels are higher in HPV-positive HNC cell lines. **(A)** HNC cell lines were lysed and analyzed by immunoblotting with the indicated antibodies. **(B)** Optical density analysis of the expression of ΔNp63α from three independent western blot experiments of HNC cell lines was performed and results were normalized to loading control (vinculin) and expressed as means ± SD. **(C)** Box plot showing the median and 10–90 percentiles of the optical density results showed in **(B)**, *P < 0.05 (unpaired *t*-test, calculated excluding UMSCC-47 value).

We screened ΔNp63α protein expression in a panel of 12 cell lines, from different subsites, divided by sex and HPV status (Citro et al., 2019). Although we used a p63 antibody able to recognize all p63 isoforms, ΔNp63α is the predominant isoform expressed in HNC (Sniezek et al., 2004). Figures 1A,B showed a clear upregulation of the protein level of ΔNp63α in HPV-positive compared to HPV negative cell lines. Among the HPV-positive cell lines, UMSCC-47 lacks the expression of ΔNp63α, due to the multiple integration of HPV16 at the TP63 locus, thus leading to the expression of a truncated 25-kDa protein at the carboxyl terminus of p63 (Akagi et al., 2014). Therefore, we excluded UMSCC-47 cells in the analysis of the distribution of ΔNp63α protein expression between HPV-positive and HPV-negative HNC cell lines. As a result, ΔNp63α protein expression is significantly higher in HPV-positive compared to HPV-negative HNC cell lines (Figure 1C).

### ΔNp63α mRNA Level Is Significantly Higher in HPV-Positive Compared to HPV-Negative HNC Cell Lines

**Figure 2:**
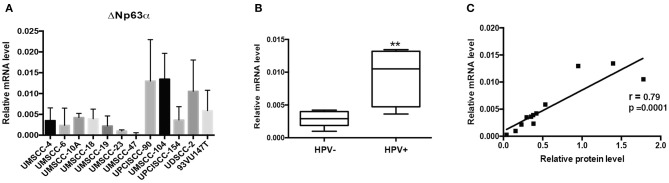
ΔNp63α mRNA levels are higher in HPV-positive HNC cell lines. **(A)** Total RNAs from HNC cell lines were isolated for RT-qPCR. ΔNp63α expression was normalized to RpP0 and expressed as means ± SD of at least three independent experiments. **(B)** Box plot showing the median and 10–90 percentiles of results showed in **(A)**, **P < 0.01 (unpaired *t*-test, calculated excluding UMSCC-47 value). **(C)** ΔNp63α mRNA levels shown in **(A)** were correlated to ΔNp63α protein expression, shown in Figure 1A, Pearson correlation coefficient *r* = 0.79 (*P* < 0.001).

We then assessed the mRNA of ΔNp63α by RT-qPCR in the same panel of HNC cell lines. As shown in Figure 2A HPV-positive HNC cell lines expressed higher levels of ΔNp63α mRNA compared to HPV-negative HNC cell lines. The same analysis performed for protein level was performed for ΔNp63α mRNA level and showed a significantly higher mRNA level in HPV- positive cell lines (Figure 2B). Moreover, as shown in Figure 2C a high correlation between ΔNp63α mRNA and protein level is present, implicating that mRNA expression may be useful in predicting ΔNp63α protein levels.

### Lack of HPV16 E6/E7 Oncoproteins Decreased ΔNp63α Expression

**Figure 3:**
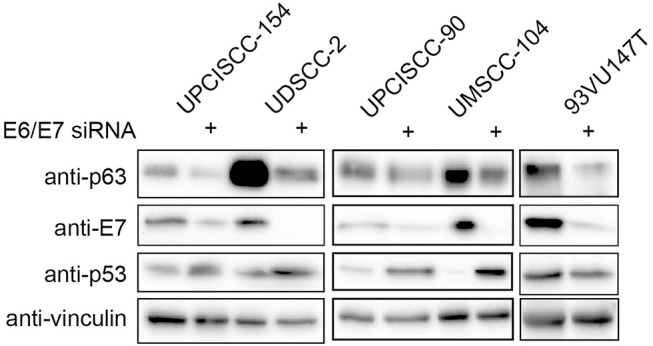
HPV16E6/E7 silencing decreases ΔNp63α expression. HNC HPV-positive cell lines were transfected with specific siRNA against HPV16E6/E7 or Luciferase as control. Seventy-two hours after transfection cells were lysed and analyzed by immunoblotting with the indicated antibodies.

We then asked whether the lack of the expression of the main HPV oncoproteins had an impact on ΔNp63α expression, by silencing of E6/E7 with specific siRNA in HPV-positive HNC cell lines. As shown in Figure 3, siRNAs were efficient in depriving the oncoproteins, also validated by p53 expression upregulation due to lack of E6. Likewise, western blot analysis revealed that ΔNp63α expression was decreasing in the absence of E6/E7, showing that ΔNp63α is E6/E7 dependent. These data demonstrate that ΔNp63α expression is E6/E7 dependent in HPV-positive HNC cell lines.

### ΔNp63α Expression Increases in HPV16E6/E7 Transformed Human Keratinocytes (HK)

**Figure 4:**
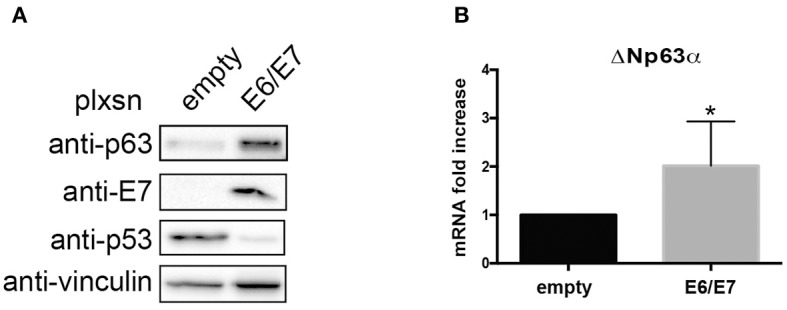
HPV16E6/E7 transduction increases ΔNp63α expression in Human Keratinocytes (HK). HK were transduced with empty or HPV16E6/E7 recombinant retroviral vectors. After selection with G418 cells were harvested. **(A)** Lysates were collected and analyzed by immunoblotting with the indicated antibodies. **(B)** Total RNAs were isolated for RT-qPCR. ΔNp63α expression was normalized to RpP0. Results from five independent experiments are expressed as means ± SD of fold changes of ΔNp63α expression of HPV16E6/E7 infected cells over control (empty vector), *P < 0.05 (unpaired *t*-test).

To further corroborate the dependency of ΔNp63α expression on E6/E7, we transduced primary HK with HPV16E6/E7 retroviral particles. Western blot analysis clearly showed an upregulation of ΔNp63α protein levels (Figure 4A). Moreover, since E6 and E7 are known to modulate the transcriptome to target diverse cellular pathway, such as cell cycle and apoptosis (Tomaic, 2016), we then investigated weather E6/E7 transduction was able to increase ΔNp63α mRNA levels. As shown in Figure 4B, E6/E7 HK had a significant higher mRNA level compared to HK control cells, suggesting that HPV16 is able to increase ΔNp63α at transcriptional level.

## Discussion

Both HPV-positive and negative tumors contain recurrent focal amplifications for 3q26/28, a region which includes squamous lineage transcription factors, such as TP63 and SOX2, as well as the oncogene, PIK3CA (Lawrence et al., 2015). However, besides genomic amplification, the TP63 gene is not frequently mutated in HNC with only a 7% mutation rate (Stransky et al., 2011) and in some cases, overexpression of p63 is likely to involve mechanisms independent of genomic alterations (Redon et al., 2001). Few studies have already shown that high risk HPV E6 and E7 oncoproteins are able to transcriptionally regulate TP63 gene, probably to facilitate the viral life cycle (Melar-New and Laimins, 2010; Mighty and Laimins, 2011; Srivastava et al., 2017). In this study we confirmed that HPV16 E6/E7 expression is able to regulate ΔNp63α transcriptionally, increasing both its mRNA and protein levels in transduced HK. Moreover, the link between HPV oncoproteins and ΔNp63α expression was confirmed in HNC HPV-positive cell lines where the lack of E6/E7 consistently decreased ΔNp63α protein levels. As a result we showed, to the extent of our knowledge for the first time, that ΔNp63α expression is significantly greater in HPV-positive compared to HPV-negative HNC cell lines, both at protein and mRNA levels. Moreover, we found a very high correlation between protein and mRNA ΔNp63α levels in HNC cell lines, suggesting that ΔNp63α protein expression can be easily predicted from quantitative mRNA data. ΔNp63α has a clear role in promoting squamous epithelial proliferation, migration, and inflammation in HNC (Rocco et al., 2006; Yang et al., 2011; Citro et al., 2019). One study reported that high p63 expression is associated with a more aggressive phenotype and poor prognosis in oral squamous cell carcinoma (OSCC) (Lo Muzio et al., 2005), whereas other studies either showed that impaired p63 expression could be important in neoplastic transformation of OSCC (Foschini et al., 2004) or could not find any significant association between p63 protein expression and survival, recurrence, or metastasis in OSCC patients (Oliveira et al., 2007). In addition, it has also been shown that high ΔNp63α protein levels accurately predict response to platinum-based chemotherapy and a favorable outcome in HNC patients (Zangen et al., 2005). Moreover, it is very well-accepted that HPV-positive HNC respond more favorably to radiation and have a more favorable prognosis compared to HPV-negative HNC (Gillison, 2004; Reid et al., 2018). Thus, further studies defining the expression of p63 in HPV-positive head and neck tumors and its association to patient survival or recurrence are needed. This might allow to use p63 as a clinical target for HPV-HNC patients, promoting the use of drugs such as HDAC inhibitors that have been shown to modulate p63 expression in HNC cell lines (Napoli et al., 2016; Citro et al., 2019).

## Data Availability Statement

The raw data supporting the conclusions of this article will be made available by the authors, without undue reservation, to any qualified researcher.

## Ethics Statement

This study was carried out in accordance with the recommendations of IEO Biobank for translational medicine (B4MED). The protocol was approved by the IEO committee. All subjects gave written informed consent in accordance with the Declaration of Helsinki.

## Author Contributions

SCi and SCh conceptualized the study. SCi, AB, AM, MS, FC, and MT were responsible for data and materials acquisition. SCi, AM, MS, and SCh worked on the data analysis and writing and editing the review.

### Conflict of Interest

The authors declare that the research was conducted in the absence of any commercial or financial relationships that could be construed as a potential conflict of interest.
